# Genome-Wide Studies of Specific Language Impairment

**DOI:** 10.1007/s40473-014-0024-z

**Published:** 2014-09-26

**Authors:** Rose H. Reader, Laura E. Covill, Ron Nudel, Dianne F. Newbury

**Affiliations:** 1 Wellcome Trust Centre for Human Genetics, University of Oxford, Oxford, OX3 7BN UK; 2 St John’s College, University of Oxford, Oxford, OX1 3JP UK

**Keywords:** Specific Language Impairment (SLI), Genome-Wide Association Studies (GWAS), Genome-Wide Linkage Analysis (GWLA), Neurogenetics, Neurodevelopmental Disorders

## Abstract

Specific language impairment (SLI) is a multifactorial neurodevelopmental disorder which occurs unexpectedly and without an obvious cause. Over a decade of research suggests that SLI is highly heritable. Several genes and loci have already been implicated in SLI through linkage and targeted association methods. Recently, genome-wide association studies (GWAS) of SLI and language traits in the general population have been reported and, consequently, new candidate genes have been identified. This review aims to summarise the literature concerning genome-wide studies of SLI. In addition, this review highlights the methodologies that have been used to research the genetics of SLI to date, and also considers the current, and future, contributions that GWAS can offer.

## Introduction

The term ‘language impairment’ (LI) encompasses a wide range of disorders that can impair, or delay, both verbal and written language abilities. Specific language impairment (SLI) is one such disorder. It occurs in 5–8 % of English-speaking school children and is an example of a verbal language impairment [1, 2]. SLI occurs despite adequate intelligence, access to education and no major neurological deficit [3]. A diagnosis is reliant on exclusion of disorders that might cause the language impairment, such as autism, cerebral palsy, hearing loss or intellectual disability [4]. The aetiology of SLI, like that of many complex neurological disorders, is not well defined, but research over the last decade has provided us with strong evidence to support the likelihood of a polygenic genetic basis [5–13, 14•]. Although it might be expected that factors such as language input would impact a child’s likelihood of developing SLI, studies have shown that most children learn to talk competently even if they are taught by adults with language impairment. This suggests that, in comparison with genes, language input has little influence on a child’s risk of developing SLI [3].

## Methodologies

Early genetic studies of SLI have been dominated by quantitative trait loci (QTL) mapping and genome-wide linkage analysis (GWLA) methodologies. Linkage studies rely on family data, aim to identify regions of the genome that are shared between related affected individuals [15] and can be either parametric or non-parametric. Parametric studies require definition of an inheritance model (e.g. dominant or recessive), penetrance and allele frequencies, while non-parametric methods are, for the most part, model free [15]. The specification of a model for SLI is not always straightforward; SLI tends to run in families and is considered to be highly heritable [16], but it only rarely follows a monogenic inheritance pattern [13]. This sporadic form of inheritance suggests that a collection of genes are at least partly responsible for the manifestation and severity of SLI in affected individuals [12]. Linkage studies may be limited in their application to complex genetic disorders because of their low resolution and pedigree limitations. Nonetheless, they can be conducted using small sample sizes and can effectively reduce the number of candidate regions to consider in preparation for targeted association analyses [17]. In contrast to linkage studies, association analyses have a high resolution and directly implicate specific genetic variants by comparing allele frequencies between cases and controls. Significantly associated variants would likely either be in linkage disequilibrium (LD) with the causal variant or be the causative variant themselves. Unlike linkage studies, association analyses assume that the causal variant will be constant across cases and will occur at a reduced frequency in controls. This can be a limiting factor, as complex disorders such as SLI are likely to involve a variable combination of variants (genetic heterogeneity), even within the same gene. In addition to case–control methods, association methods can be applied within family-based cohorts by comparison of allele frequencies between probands and unaffected family members [13].

## SLI GWLA Studies

Two initial GWLA studies of SLI identified novel candidate regions that are linked to performance on tests of language-related ability, in families affected by SLI [5–7]. The first study, conducted by the SLI Consortium (SLIC), identified two novel susceptibility loci on chromosomes 16q23–24 (SLI1, OMIM 606711) and 19q13 (SLI2, OMIM 606712) [6]. A subsequent study by Bartlett et al. highlighted 13q21 (SLI3, OMIM 607134) and 2p22 as additional loci predisposing to SLI [7].

SLIC analysed 98 UK families (473 individuals), each with a proband whose language scores fell ≥1.5 standard deviation (SD) below the mean for their age and nonverbal IQ scores that were within the specified normal range (>80). This study considered three quantitative language measures derived from the Clinical Evaluation of Language Fundamentals—Revised (CELF-R) and non-word repetition (NWR) [6]. CELF-R is a commonly used test battery designed to assess both receptive and expressive language ability in school-age children [18]. The NWR test measures the ability to retain novel phonological (speech sound) information for short periods of time; this is commonly impaired in people with SLI [5, 11]. Following the language tests, linkage analysis was performed using 400 highly polymorphic microsatellite markers. Significant linkage was detected on chromosome 16 with the NWR trait (LOD score 3.55; *P* = 0.00003) and on chromosome 19 with the CELF-R expressive language score [ELS] (LOD score 3.55; *P* = 0.0004) [6].

Bartlett et al. analysed five Canadian families (including 73 individuals) of Celtic ancestry. Individuals were sorted into three categorical groups, labelled ‘language-impaired’, ‘reading-impaired’ and ‘clinically impaired’ on the basis of their performance across six quantitative language tests. Language-impaired individuals were defined by a Spoken Language Quotient (SLQ) score (from the Test of Language Development) [19] of ≤85, reading-impaired individuals had a non-word reading and IQ discrepancy, and clinically impaired individuals had a history of speech and/or reading therapy [7, 8]. Linkage to region 13q21 was detected under a recessive model in the reading-impaired group (LOD score 3.92; *P* < 0.01), and linkage to 2p22 was detected under a recessive model of the language-impaired group (LOD score 2.86; *P* < 0.06) [7].

The lack of overlap between the SLI susceptibility regions, implicated by these two independent linkage studies, not only supports the theory of locus heterogeneity but also demonstrates the statistical complexity of replicating genetic loci in different cohorts with variable phenotypes. Susceptibility regions 13q21 and 2p22, highlighted by Bartlett et al., may not have been detected by SLIC, because of alternative allele frequencies within the UK sample set. Although the Canadian families in the study by Bartlett et al. were not considered population isolates, they were selected from a different ethnic background compared with that of the SLIC cohort, and thus the markers carried in these regions may have been elevated to a detectable linkage peak in this group. Furthermore, SLIC and Bartlett utilised slightly different linkage methodologies and diagnostic criteria for determining SLI affection status. SLIC diagnosed probands on the basis of a clinical verbal-language battery [5, 6], whereas Bartlett included a more varied set of phenotypes, including reading ability (designed to reflect the proband’s overall language ability), and then classified all individuals as being affected or unaffected under three alternative definitions [7, 8]. SLIC used non-parametric linkage methods, whereas Bartlett used parametric linkage, assuming 7 % population penetrance, and used both dominant and recessive models of inheritance [5–8].

Despite the inconsistency of loci linked to SLI, both SLIC and Bartlett have since replicated their findings [5, 8]. SLIC conducted a targeted linkage study in 2004, with an additional 86 nuclear families selected and characterised as described for the SLIC samples above. Linkage was detected again on chromosomes 16 (LOD score 2.86; *P* < 0.02) and 19 (LOD score 2.31; *P* < 0.02), both to the NWR trait. The two SLIC cohorts were then pooled to total 184 families and 840 individuals. In this pooled dataset, highly significant linkage was detected on chromosome 16 [5].

In 2007, SLIC applied a genome-wide multivariate linkage approach to their pooled cohort, which was able to analyse linkage to multiple quantitative traits simultaneously. In total, they investigated 11 measures of spoken and receptive language ability, reading ability and non-verbal IQ. This study supported the Consortium’s previous evidence linking loci on chromosomes 16q (*P* = 0.008) and 19q (*P* = 0.017), and highlighted a novel region of linkage on chromosome 10q26 (*P* = 0.019) [9]. The linkage on chromosome 16q was specific to NWR in the previous univariate SLIC studies, and in the multivariate study it was linked to NWR and literacy measures (single-word reading and single-word spelling) [5, 6, 9], indicating that variation in this region will likely impact phonological memory. An inability to store short-term verbal information is likely to impair the ability of an individual to acquire and retain language skills, and this has been a growing, aetiological theory surrounding SLI [20, 21]. In contrast, linkage to chromosome 19q had previously been detected with multiple traits, firstly to ELS [6], then to NWR [5], and in the multivariate study it was found to be linked to a variety of expressive and language traits [9]. This suggests that the risk variants within this linkage region may impact upon a variety of language abilities.

One final study further expanded the SLIC cohort, analysing an additional 300 individuals from 93 families affected by SLI, and again replicated linkage of chromosome 16q (*P* = 0.002) with NWR, and linkage of chromosome 19q (*P* = 0.007) with ELS [22].

Bartlett et al. also expanded their cohort to include 22 US-based nuclear families with at least one individual per family affected by SLI, in combination with the original families studied [8]. In total, 365 DNA samples were genotyped for microsatellites on chromosomes 2 and 13, enabling replication of the chromosome region 13q21 linkage, using similar parametric modelling procedures. Further analysis revealed that only a small percentage of the families contributed to this linkage, suggesting that the risk factor is not sufficient or necessary for SLI to manifest itself in all families. In this combined sample set, the linkage region on chromosome 2 could not be replicated, suggesting that if SLI risk factors exist on chromosome 2, they may have a small effect size with low penetrance, which would make them difficult to identify using a linkage study [8].

In addition to the SLIC and Bartlett studies, a few other groups have also investigated the genetic basis of SLI, using single families and isolated populations [12, 23, 24, 25•]. These studies provided a unique opportunity to look at individuals with an increased level of shared environmental and genetic influences. When certain phenotypes become more prevalent in isolated populations, it may suggest that a founding genetic influence has been shared amongst the group and may thus be more common and somewhat easier to identify.

A classic linkage study, conducted prior to the two described above, linked language impairment to a region designated SPCH1 on chromosome 7q [26, 27]. This study analysed a single, large, three-generation pedigree known as the KE family, in which approximately half of the individuals were affected with a severe speech and language disorder. GWLA identified a region on chromosome 7q31 (maximum LOD score 6.62) that co-segregated with the language disorder in this particular family [26] and has since been narrowed down to a causative mutation in the *FOXP2* (forkhead box P2) gene (OMIM 605317) [24]. It is important to note that not all members of the KE family would meet the selection criteria for studies of *specific* language impairment, because they had evidence of both intellectual disability and motor impairment. In addition, the severe language impairment exhibited by members of the KE family surpasses the typical SLI phenotype, in the sense that it involves a variety of associated neurological dysfunctions. All of the *FOXP2* coding regions, or exons, have since been screened for association with SLI, using 43 SLIC probands, but no associations or mutations were detected in this study [28]. It is likely, then, that the *FOXP2* gene remains functional in typical SLI probands. Despite this, the *FOXP2* gene is clearly vital for language acquisition, as demonstrated by the KE phenotype [26]. The KE phenotype has since been described as childhood apraxia of speech (CAS), which has been linked to disruptive variants within *FOXP2*, as demonstrated by similarly affected families [29–32]. Subsequent studies of CAS also identified 16 submicroscopic deletions and duplications (copy number variants [CNVs]) in half of the participants [33]. These fell across ten different chromosomes and have the potential to cause disruptions in speech and language development. Of note, overlapping deletions at chromosome 16p13.2 were found in two of the participants, though the region does not overlap with previously implicated loci on chromosome 16, and the phenotypes associated with CNVs in this region have not been characterised [33]. High-throughput sequencing methods have also been applied to individuals affected by CAS [34]. Although this study sequenced the entire exomes (i.e. all known gene-coding regions) of ten CAS probands, it reported only mutations that affected known candidate genes. The study reported potentially clinically relevant variants (i.e. those variants that were predicted to have a deleterious effect upon protein function and had a reported population frequency of <0.3 %) in eight of the ten individuals investigated. These were distributed across six candidate genes that had previously been associated with CAS (*FOXP1* [forkhead box P1], *CNTNAP2* [contactin associated protein-like 2]) or overlapping phenotypes (*ATP13A4* [ATPase type 13A4], *CNTNAP1* [contactin associated protein 1], *KIAA0319* and *SETX* [senataxin]) but did not include any *FOXP2* mutations [34]. Although preliminary, the findings of this study suggest that the application of high-throughput methodologies and comprehensive analyses of the arising data may prove fruitful in future studies of speech and language impairments.

In 2010, a linkage study was conducted on a three-generation German family, the NE family, with multiple members affected by variable language and literacy impairments [23]. Psychoacoustic tests demonstrated an auditory processing deficit that co-segregated with these impairments. The investigators hypothesised that this deficit disallowed the affected family members to discriminate between tone durations, putting them at an increased risk of language-related disorders. Linkage analysis suggested that a large 58.5 Mb region, containing 600 genes, on chromosome 12p13.31–12q14.3 may contain a contributory variant, but the specific variant has yet to be identified [23].

Another independent linkage study investigated an isolated Chilean population with an increased prevalence of SLI. A series of language tests indicated that ~35 % of the island’s children met criteria for SLI, and a further 27.5 % had impaired language skills accompanied by other neurological deficits [12]. Parametric and non-parametric linkage analyses found consistent linkage to a 48 Mb stretch of chromosome 7q31–q36 (LOD score 6.73; *P* = 4.0 × 10^−11^), which included the genes *FOXP2* and *CNTNAP2* (OMIM 604569) [12], the significance of which is discussed later in this article. No single co-segregating chromosome regions were identified using parametric linkage analyses, which supports the likelihood of a polygenic aetiology of SLI in this population.

A recent linkage study detected a heterozygous 4 kb deletion at chr2q36 (SLI5; OMIM 615432) in 15 Southeast Asian probands with language delay and white-matter hyper-intensities (WMH), which are common markers of aging [25•]. The deletion, which eliminated exon 3 of the *TM4SF20* [transmembrane 4 L six family member 20] gene, co-segregated with language delay in the 15 families studied and appeared to represent an ancestral haplotype confined to Southeast Asian populations, notably Vietnamese, Thai and Burmese, with an allele frequency of ~1 % [25•]. The function of the *TM4SF20* gene is unknown, and its function has yet to be assessed in other SLI populations.

Another study described a geographically isolated, Russian-speaking population with an increased prevalence of SLI [35]. The settlement involves ~871 people, 20–40 % of whom have language impairment [35]. At present, no genetic studies have been conducted using this population, but it is likely that the increased prevalence of SLI is caused by a founder mutation that is now widespread amongst the settlers, as seen in previous family and isolated-population studies of language impairment [12, 23, 25•].

Populations with an increased prevalence of language impairment can assist with the identification of candidate genes. It is assumed that the causative variant will be found more commonly within the affected population and will thus be easier to associate with the impairment. Future studies would benefit from investigating the role of the candidate genes that have been identified in these populations.

## SLI Targeted Association Studies

Several studies have suggested shared genetic aetiology of speech disorders and reading disability (RD)—in particular, Speech Sound Disorder (SSD)—which is distinct from SLI but is rarely treated as such. SSD is considered to be a problem associated with phonological awareness, which leads to a deficit in age-appropriate speech sound production [36].

Targeted linkage studies have identified significant linkage for SSD within RD candidate regions. These include chromosomes 6p22, 15q21 (DYX1; OMIM 127700), 1p36 (DYX8; OMIM 608995) [36] and 3p12–q13 (SSD; OMIM 608445 and DYX5; OMIM 606896) [37]. A more recent study carried out GWLA in a large pedigree, exhibiting familial SSD and a motor sequencing deficit. The study identified linkage peaks on chromosomes 6p21, 7q32, 7q36 (overlapping with SLI4) and 8q24 primarily with the motor sequencing phenotype, and on 7q31.q32–34 with SSD [38].

A recent study also investigated whether autism and SLI share genetic risk factors, by conducting linkage analysis in 79 families affected by SLI and/or autism. Two linkage peaks were found on chromosomes 15q23–26, relating to oral language ability, and 16p12, relating to written language ability, within the subset of families that included individuals with both autism and SLI. However, neither linkage peak was independently associated with one disorder, indicating that each locus influenced both SLI and autism [39•].

Although the numbers of studies in this field are relatively small and consider only targeted regions, the findings indicate a high level of heterogeneity and the involvement of many contributory factors in language disorders. Following the discovery of SLI linkage regions, the subsequent targeted studies in this field aimed to narrow down those broad regions to allow identification of specific risk variants. Targeted studies have the advantage of investigating smaller regions with greater marker density, and therefore resolution, making them more powerful in detecting linkage and association.

A targeted association study, focusing on the chromosome 16q region [5, 6, 9, 22], pinpointed candidate genes for SLI [11]. The SLI1 region was screened using 1906 single nucleotide polymorphisms (SNPs) across 58 genes. Two clusters of variants were significantly associated with NWR and fell within *CMIP* (c-maf-inducing protein) at rs6564903 (*P* = 5.5 × 10^−7^) and *ATP2C2* (calcium-transporting ATPase type 2C) at rs11860694 (*P* = 2.0 × 10^−5^). These findings suggest that *CMIP* and *ATP2C2* are somehow involved in developing, or retaining, phonological short-term memories, which may be important for language acquisition [11]. Note that since the function of the identified variants remains unknown, it is possible that their effects are exerted through transcripts other than *ATP2C2* and *CMIP*.

In addition, a study by Lonardo et al. presented a patient with postural, motor and speech delay, and severe learning and behavioural difficulties [40]. They established that the patient possessed an inverted de novo tandem duplication of 16q22q11.2, although they could not establish a true correlation of genotype to phenotype, because of the size of the duplication [40]. The findings of this study support the implications that a region of chromosome 16 plays an important role in the development of speech and language. Although the duplication at 16q22q11.2 that was observed in this study does not include *CMIP* (16q23.2) and *ATP2C2* (16q24.1), it does suggest that chromosome 16 may include a number of genes that influence the development of language [5, 6, 9, 11, 22, 40].

An additional gene on chromosome 7q35–q36, known as *CNTNAP2* (which is down-regulated by *FOXP2* [10]), has also been associated with SLI. A functional study identified a *CNTNAP2* fragment that was bound by the FOXP2 protein, regulating its expression. A targeted association analysis in the SLIC cohort identified significant associations between NWR and nine intronic SNPs in *CNTNAP2* [10]. A subsequent association study supported the link between variants within the same region of *CNTNAP2* and language ability in a general population sample [41].

Recently, a targeted association study considered the role of the HLA region on chromosome 6p, which is important for the function of the immune system, in SLI [42]. Among the genes implicated in the study, which included both quantitative and case–control analyses, were *HLA*-*A* (major histocompatibility complex, class I, A), *HLA*-*B* (major histocompatibility complex, class I, B) and *HLA*-*DRB1* (major histocompatibility complex, class II, DR beta 1), which were previously implicated in autism [43], attention-deficit/hyperactivity disorder (ADHD) [44, 45] and schizophrenia [46].

In summary, five regions have consistently been associated with SLI, using GWLA, and targeted association studies have enabled identification of risk variants and candidate genes within three of these regions [13, 47] (see Table 1).

**Table 1:** A summary of specific language impairment (SLI) linkage in OMIM

Region	Cytogenetic loci	Associated gene(s)	OMIM no.	Method	References
SLI1	16q23.1–16q24	*CMIP*; *ATP2C2*	606711	GWLA, targeted association	[4, 5, 10]
SLI2	19q13.13–19q13.41	N/A	606712	GWLA	[4, 5]
SLI3	13q14.3–13q31.1	N/A	607134	GWLA	[6, 7]
SLI4	7q31–7q36	*CNTNAP2*	612514	GWLA, targeted association	[4, 5, 9]
SLI5	2q36	*TM4SF20*	615432	LA	[25•]

*ATP2C2* calcium-transporting ATPase type 2C, *CMIP* c-maf-inducing protein, *CNTNAP2* contactin associated protein-like 2, *GWLA* genome-wide linkage analysis, *LA* linkage analysis, *N*/*A* not applicable, *TM4SF20* transmembrane 4 L six family member 20

More recent publications in the language impairment field have begun to incorporate high-throughput SNP bead/chip assays to conduct genome-wide association studies (GWAS) of SLI and related traits [14•, 48, 49].

## GWAS of Language Impairment

GWAS offer a high-resolution scan of a growing number of consistently variable loci within the genome and have been empowered by the development of genetic variation databases, such as the HapMap project [50] and the 1000 Genomes Project [51]. GWAS are conducted using SNP arrays and compare the frequencies of hundreds of thousands common di-allelic SNPs between affected and unaffected individuals. Under the common-disorder, common-variant hypothesis, accumulatively, these common SNPs may predispose individuals to various genetically complex diseases, including SLI. Thus GWAS methods are an ideal, and cost-effective, way of pinpointing possible risk variants.

Compared with other neurodevelopmental disorders, relatively few GWAS have been performed for SLI. Traditional case–control association studies typically require thousands of cases and controls [52] to gather adequate power, but at present no SLI cohort reaches this size. It may be possible to employ family-based association methods, which can use information from parents as well as probands and may provide increased power. For example, Weinberg et al. developed a method that uses case–parents trios and obtained moderate power when testing for child genetic effects using 100 case–parents trios and high power when testing for parent-of-origin effects (with effect sizes ranging between 2 and 3) [53]. In addition, despite being underpowered, smaller cohorts may still detect true associations that contribute across disorder populations [54]. A recent study performed a GWAS using the SLIC cohort [14•]. This study used family subsets (e.g. case–parents trios) in a multinomial association method which compares the likelihoods of null and risk models [55]. This approach allows for greater power when using parents and offers flexibility in the type of analysis performed. This particular study considered parent-of-origin effects, in addition to the more commonly considered child genotype model, and used quantitative language measures to assign SLI affection status. The authors found genome-wide significant paternal parent-of-origin effects with a SNP in *NOP9* [nucleolar protein 9] (*P* = 3.74 × 10^−8^) on chromosome 14q12 and suggestive maternal parent-of-origin effects on chromosome 5p13 (*P* = 1.16 × 10^−7^) in a linkage region previously implicated in autism [56] and ADHD [57]. The *NOP9* gene codes for an RNA-binding protein [58], which was shown to be dysregulated in schizophrenia patients [59]. The Avon Longitudinal Study of Parents and their Children (ALSPAC) population cohort was used to replicate the maternal association on chromosome 5; paternal genotypes were unavailable (*P* = 0.001), albeit in the opposite direction (i.e. the risk allele was different).

As discussed above, children with SLI often have other language disorders, or learning disorders, such as RD, SSD, ADHD or autism, which suggests that there may be an overlap of genetic risk factors [13, 60–62]. It may therefore be beneficial to combine cohorts to investigate language disorders together and increase the sample sizes. In a study of educational attainment, Rietveld et al. demonstrated that the size of a cohort can outweigh a strict phenotype selection in defining the underlying genetic aetiology of a disorder [63]. In a similar approach, more recent language impairment studies have utilised GWAS methods to investigate overlapping genetic risk factors between learning disorders.

## GWAS Involving Disorders that Co-occur with SLI

Two GWAS of language-related traits in population samples have been reported. The first examined reading and language-related traits across the entire range in two independent population cohorts (the Brisbane Adolescent Twin Sample [BATS] and ALSPAC) [64] and then went on to perform a meta-analysis. The most significant association was with a SNP in *ABCC13* [ATP-binding cassette, sub-family C (CFTR/MRP) member 13] (*P* = 7.34 × 10^−8^ in the combined cohort) on chromosome 21q11.2. *ABCC13* (OMIM 608835) is thought to be a pseudogene that used to be involved with transporting molecules across intra- and extracellular membranes [64]. A second study also used the ALSPAC cohort but selected individuals for low language and/or reading skills from this sample set and compared allele frequencies within these subsets with the rest of the ALSPAC distribution [48]. The authors reported associations when considering individuals with concurrent RD and language impairment in *ZNF385D* [zinc finger protein 385D] (*P* = 5.45 × 10^−7^) on chromosome 3p24.3 and *COL4A2* (collagen, type IV, alpha 2) [OMIM 120090] (*P* = 7.59 × 10^−7^) on chromosome 13q34. When examining individuals with only language impairment, the strongest association was reported for a SNP on chromosome 4q26, in *NDST4* (N-deacetylase/N-sulfotransferase (heparan glucosaminyl) 4) [OMIM 615039] (*P* = 1.4 × 10^−7^).

Very recently, Gialluisi et al. conducted a GWAS meta-analysis involving individuals with both reading and language impairments [65]. This study included 1,862 individuals from families affected by RD or SLI in the UK Reading Disability (UK-RD), SLIC and Colorado Learning Disabilities Research Centre (CLDRC) databases. A family-based GWAS was performed on sibling pairs from each of the three datasets before combining all of the samples and conducting a meta-analysis. Two SNPs, rs59197085 on chromosome 7q32.1 and rs5995177 on chromosome 22q12.3, were found to be associated at a suggestive level (P ≈ 10^−7^) with a range of reading and language traits [65]. rs59197085 falls within the *CCDC136* (coiled-coil domain containing 136) gene and falls within the SLI4 region of linkage. rs5995177 lies within the *RBFOX2* (RNA-binding protein, fox-1 homolog 2) gene, which is heavily expressed in the neurons of the developing foetal brain [66]. *RBFOX2* has been implicated in many neurological disorders, including Rolandic epilepsy [67] and autism [68]. In addition, *RBFOX2* is both a FOXP2 target [69] and part of a cascade of genes that interact with the *DYX1C1* (dyslexia susceptibility 1 candidate 1) gene [70, 71]. This study did not replicate the associations found in *ATP2C2*, *CMIP* or *CNTNAP2*. This is likely due to the inclusion of multiple affection phenotypes—language, reading and learning disability [65]. The combination of all of those phenotypes would likely increase genetic heterogeneity and lead to weaker associations with specific components, although the authors sought to overcome this limitation by implementing a principal component approach.

## Conclusion

**Fig. 1:**
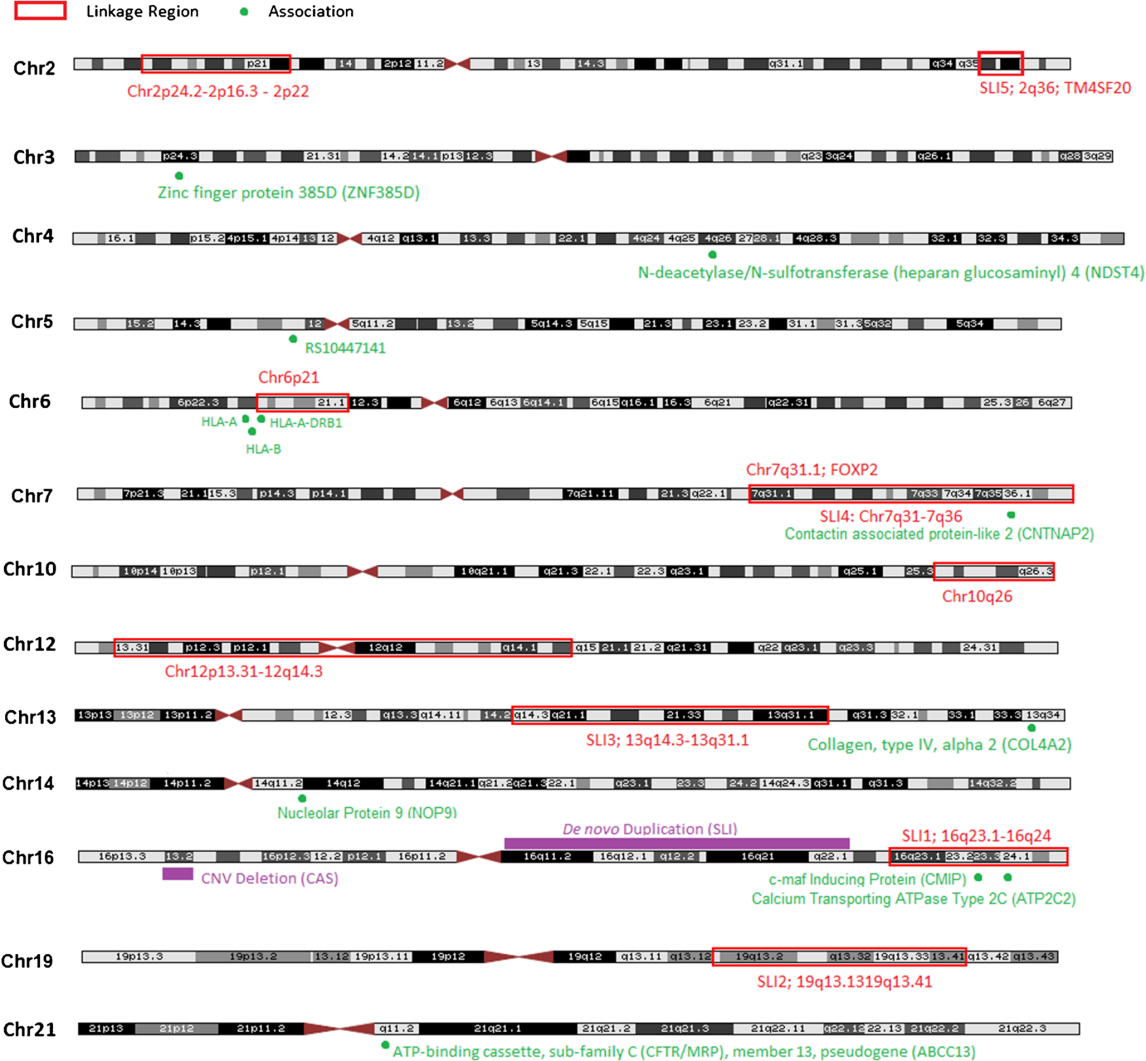
A diagrammatic representation of specific language impairment (SLI) linkage regions and associations

Linkage and association studies have highlighted a number of genetic regions that may predispose individuals to SLI, as summarised in Table 1. Targeted association studies have been particularly successful in narrowing linkage regions into candidate genes, of which there are now four to consider (Table 1). More recently, GWAS have been used to identify SNPs that might contribute to language ability or predispose individuals to language impairment, successfully highlighting additional genes for consideration. However, there is currently very little overlap in the findings between these various studies (Fig. 1). Future studies concerning SLI would benefit from functional studies, determining the role of candidate genes and risk variants that have been identified to date. These studies will allow us to identify exactly how the risk variants predispose individuals to language impairment and help elucidate the pathways underlying such complex disorders. In addition, it is clear that future studies will benefit from larger sample sizes, clearer definitions of SLI, more consistent selection criteria and investigation of related phenotypes. Neuroimaging studies, for example, have been very successful in identifying brain regions that are important for language and are noticeably altered in language-impaired individuals [72–74]. Such biological markers may prove useful as endophenotypes, representing altered brain activation patterns and structural volumes that correlate with language impairment and RD candidate gene dysfunction [48, 75]. It is also likely that we will start to see data from whole-genome and exome sequencing studies that will complement existing linkage and association studies, as evidenced by preliminary studies in CAS [34]. Sequencing methods can offer greater power to detect rare and common variants, even if they have only small effect sizes, but still require large sample sizes and careful controls for reliable identification of candidate genes [76]. Ultimately, in combination with the other methodologies and criteria described in this article, whole-genome and exome sequencing will assist with the identification of novel candidate genes and variants that may contribute to the risk of developing SLI; this may lead to earlier clinical intervention. The combination of data and expertise across these diverse disciplines will enable better understanding of the biological basis of language impairment.
